# The impact of industrial agglomeration on urban green land use efficiency in the Yangtze River Economic Belt

**DOI:** 10.1038/s41598-023-28250-7

**Published:** 2023-01-18

**Authors:** Jingtong Wang, Ping Han

**Affiliations:** School of Economics, Harbin University of Commerce, Harbin, 150028 Heilongjiang People’s Republic of China

**Keywords:** Ecology, Ecology, Environmental sciences, Environmental social sciences

## Abstract

At present, the insufficient supply of land resources has seriously hindered the sustainable development of regional economy. Improving the urban green land use efficiency (UGLUE) has become a key issue on the road to sustainable development. As an important feature of economic development, industrial agglomeration has an impact on the UGLUE that cannot be ignored. This paper uses the Global Malmquist–Luenberger Index (GMLI) to measure UGLUE of 107 cities in the Yangtze River Economic Belt (YREB) from 2007 to 2016, and uses a dynamic panel model (DPM) to empirically analyze the effects of industrial specialization agglomeration and diversification agglomeration on UGLUE. On this basis, the heterogeneous impact of industrial agglomeration in different regions on UGLUE is further discussed. The results illustrate that: (1) The UGLUE shows a general downward trend. (2) Different modes of industrial agglomeration have different impacts on UGLUE. The impact of industrial specialization agglomeration on UGLUE was inverted U-shaped. Industrial diversification agglomeration has a positive effect on UGLUE. (3) The impact of industrial agglomeration in different regions on UGLUE is heterogeneous. The relationship between the industrial agglomeration and UGLUE in the YREB revealed in this paper will provide a reference for promoting UGLUE.

## Introduction

As the spatial carrier of economic and social activities, urban construction land plays a vital role in promoting economic development^1^. With the rapid economic development, urban construction land has begun to expand rapidly^2^. A series of problems such as large loss of ecological land, heat island effect and environmental pollution have appeared^3^, which restrict the sustainable development of region to a large extent^4^. In order to solve these problems, China introduces the most stringent land use system, which strictly control the use of incremental land and prohibit the arbitrary expansion of urban construction land area. In this context, the improvement of urban green land use efficiency (UGLUE) become the key to solving the contradiction between the shortage of land resource and economic growth, promoting regional sustainable development^5^.

Land use is affected by many factors such as economic development level^6^, industrial structure upgrading^7^, technological level^8^, globalization, marketization, decentralization and urbanization^9^ and so on. Among these factors, industrial agglomeration is an important way to promote effective land use^10^. It can promote the rational use of elements through the spillover of knowledge and technology, flow of human capital and sharing of infrastructure^11^. Industrial agglomeration includes two types of agglomerations: industrial specialization agglomeration and industrial diversification agglomeration^12^. Because the externalities brought by two types of agglomeration are different^13^, it is necessary for us to analyze the heterogeneous effects of different industrial agglomeration modes on UGLUE.

The Yangtze River Economic Belt (YREB) covers 11 provinces and cities in China, which has a huge number of cities^14^. Compared with other regions, the urban construction land resources in the YREB are more scarce. The contradiction between insufficient supply of land resources and economic growth is more prominent. As the region with the strongest overall strength in China, the improvement of UGLUE in the YREB not only effectively solves the contradiction between the shortage of land resources and economic growth in the YREB, but also provides a reference for other cities to solve land use problems.

Based on this, taking 107 cities in the YREB as the research object, this paper discusses the impact of different industrial agglomeration modes on UGLUE and analyzes the heterogeneity of the impact of industrial agglomeration in different areas on UGLUE. Finally, from the perspective of industrial agglomeration, this paper puts forward relevant policy suggestions on promoting the effective use of urban land.

The contributions of this paper are as follows: Firstly, the GMLI is used to measure UGLUE. This method not only takes the input factors such as capital, labor, and land consumption into account, but also analyzes the dynamic changes of UGLUE. Secondly, from both theoretical and empirical aspects, this paper analyzes the difference of the impact of industrial specialization agglomeration and industrial diversification agglomeration on UGLUE, which makes the policy suggestions on improving UGLUE of the YREB more targeted. Thirdly, this paper selects 107 cities in the YREB as the research objects, providing new empirical evidence for the research on the relationship between industrial agglomeration and UGLUE.

## Literature review

In recent years, the issue of land use efficiency has received extensive attention from scholars. In terms of definition, different fields have different definitions of land use efficiency. From an industrial perspective, land use efficiency is defined as the level of industrial output per unit of industrial land area^3^. From an agricultural perspective, land use efficiency is defined as the crop yield per unit of farmland. In this paper, the definition of UGLUE is the average economic output per square kilometer of urban construction land under the constraint of undesired output^15^. In terms of measurement methods, some scholars used the stochastic frontier analysis model to measure land use efficiency^16^. Some scholars also use data envelopment analysis (DEA) model to measure land use efficiency^17^. Compared with stochastic frontier analysis, DEA model does not need to set a specific function form and is easy to operate. In order to reflect the influence of unexpected output, slacks-based measure model and directional distance function model considering unexpected output are gradually used^18,19^.

Regarding the influencing factors of land use, some scholars have pointed out that there is a relationship between macroeconomic development and land use that cannot be ignored. For example, based on the Wuhan urban agglomeration, Gao et al.^7^ found that the economy could improve urban land use efficiency by promoting the optimal allocation of resources. Masini et al.^20^ found that richer cities have higher land use efficiency. Through regression analysis, Yu et al.^8^ found that economic development can effectively improve land use efficiency. Some scholars believe that land use also depends on government intervention^21^. As Brueckner^22^ pointed out, the governmental intervention in the land market caused the results of land use to be very different from those of free market. In addition to the two influencing factors of macroeconomics and governmental intervention, population density and urbanization all have an impact on land use^23,24^. Land is an important economic factor, and its input–output efficiency is closely related to industrial agglomeration. However, there are a few studies on the relationship between industrial agglomeration and land use.

As an important economic factor, the input–output efficiency of land is closely related to industrial agglomeration. However, there are few researches on the relationship between industrial agglomeration and land use. At present, the research on industrial agglomeration mainly focuses on the relationship between industrial agglomeration and economic growth, labor productivity and environmental pollution. Several studies have documented a positive relationship between industrial agglomeration and economic development. Zheng et al.^25^ found that when agglomeration reached a certain level, industrial agglomeration would have a positive impact on the improvement of industrial energy efficiency. Han et al.^26^ pointed out that specialization and diversification clustering would significantly reduce the energy efficiency of neighboring cities. Tanaka et al.^27^ found that the industrial agglomeration effect of paper and pulp industry in Japan had a positive impact on energy efficiency. Najkar et al.^28^ believes that industrial agglomeration can promote the improvement of production efficiency of Iran's food manufacturing industry. By studying the plastics industry in Colombia, Bernal^29^ found that industrial specialization agglomeration could generate economies of scale and reduce production costs of enterprises. However, some empirical studies also show the negative effects of industrial agglomeration. According to Dong et al.^30^, industrial agglomeration at the national level will intensify pollution agglomeration. Zhao et al.^31^ made use of provincial panel data of China's textile industry and found that when the degree of industrial agglomeration is low, promoting industrial agglomeration can improve energy efficiency. However, when industrial agglomeration reaches a certain level, it is negatively correlated with energy efficiency.

The above literature provides reference for the research on the impact of industrial agglomeration on the UGLUE, but there are still the following defects: Firstly, most of the existing literatures use a single index to measure UGLUE, which leads to errors in the measurement of UGLUE. Secondly, the existing literature has analyzed the influence of industrial agglomeration unilaterally, and little literature has analyzed the positive and negative influences of industrial agglomeration theoretically, and compared the causes of different influences. Finally, there is no literature focusing on the YREB to analyze the relationship between industrial agglomeration and UGLUE in the YREB.

## Research hypothesis

In this paper, UGLUE is defined as: under the constraint of land resource scarcity, and based on the principle of minimizing environmental pollution, the unity of economic benefits, social benefits and ecological benefits is realized. Its connotation is mainly manifested in three aspects: firstly, economic connotation. To get more economic output by putting in the least amount of land if other conditions are met. Secondly, social connotation. The use of urban land should meet the material and spiritual needs of residents and improve their happiness. Thirdly, ecological benefits. In the process of urban land use, reduce the emission of pollutants to reduce the damage to the environment.

Industrial agglomeration refers to the process that some industries carry out concentrated production and sales activities in a certain space to promote the rapid flow of various factors and resources in the space. Industrial agglomeration mainly includes two forms: industrial specialization agglomeration and industrial diversification agglomeration. Industrial specialization agglomeration refers to the agglomeration of the same or similar industries. This form of industrial agglomeration can promote the exchange of information and technology between different enterprises in the same industry and improve production efficiency. Industrial diversification agglomeration refers to the agglomeration of various types of industries. Different from industrial specialization agglomeration, industrial diversification agglomeration can promote knowledge and technology exchange among different industries, so as to make all production links of products closer, reduce production costs and improve production efficiency. Due to the different characteristics of industrial specialization agglomeration and industrial diversification agglomeration, two agglomeration modes should be taken into consideration in the study of industrial agglomeration, so as to more accurately analyze the impact of industrial agglomeration on UGLUE.

### Industrial specialization agglomeration and UGLUE

**Figure 1:**
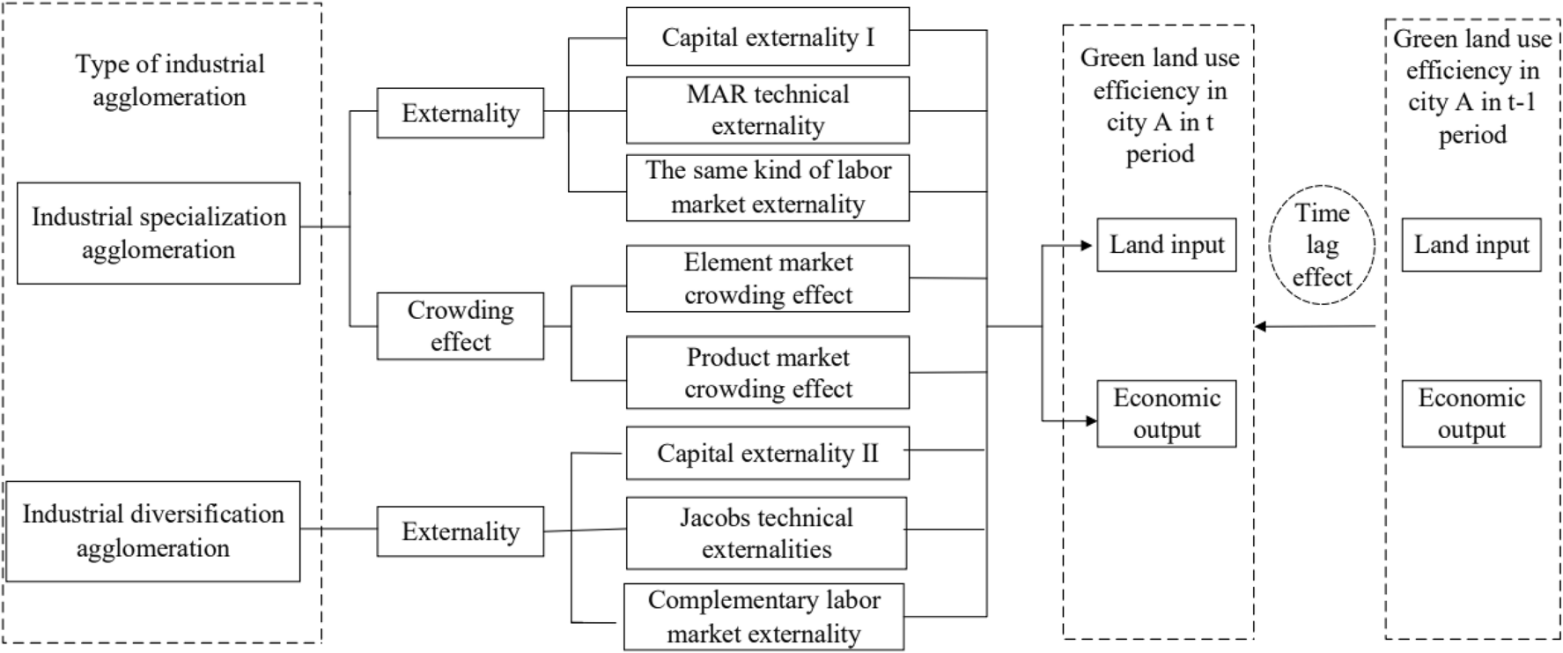
The impact mechanism of industrial agglomeration on UGLUE.

The mechanism of industrial specialization agglomeration on UGLUE is complicated (see Fig. 1). On the one hand, industrial specialization agglomeration generates externalities, thereby promoting the improvement of UGLUE. Externalities are mainly manifested in three aspects: Firstly, capital externalities. There is a vertical production structure among similar enterprises. The upstream and downstream enterprises are closely connected. Capital factors flow from low-productivity companies to high-productivity companies. High-productivity companies obtain capital investment during expansion more easily, thereby achieving effective configuration of element. We call this kind of capital externality as capital externality I. Secondly, technical externalities. A large concentration of a certain industry in a specific space promote the spillover of knowledge and technology in the industry, allowing similar enterprises to learn from each other, thereby improving production efficiency. The externality generated by knowledge split among similar enterprises is called MAR technical externality. Thirdly, the externalities of the labor market. The large concentration of similar industries in specific areas makes it easier for labor to find similar jobs, which not only reduces the cost of workers learning skills in other industries, but also reduces the cost of corporate recruitment and training, enabling companies to allocate more funds used for technology development, we call this externality as the same kind of labor market externality. The positive externalities generated by industrial specialization agglomeration increase the economic benefits of land, thereby improving UGLUE.

On the other hand, constrained by the scarcity of land resources, industrial specialization agglomeration has crowding effect, which inhibits the improvement of UGLUE. The crowding effect is mainly reflected in the following two aspects: Firstly, the crowding effect of factor markets. When the number of similar enterprises reaches saturation, enterprises begin to compete for production factors. The demand for production factors increases and the prices continue to rise, which increase the production costs of enterprises. The enterprise have to adopt a low-price selling strategy for products. Secondly, the crowding effect of product market. A large concentration of similar enterprises form a strong production capacity. As the market demand remains unchanged, the increase in supply cause fierce competition among enterprises in product market. Enterprises begin to compete to reduce prices of products, which form malicious competition. The crowding effect of factor and product markets severely squeeze corporate profit margins and reduced corporate production efficiency.

The agglomeration of similar industries in a certain scale reduces the production cost, improves the production benefit of enterprises and the economic benefits of land through capital externalities, technological externalities and similar labor market externalities. However, with the expansion of agglomeration scale, the crowding effect begins to take shape. The companies begin to conduct malicious competition in the factor market and product market, which reduces the production efficiency. In recent years, due to the attraction of agglomeration externalities, the industrial structure of YREB shows a trend of homogeneity. A large number of similar industries congregate in a specific area. Enterprises are oversaturated. The crowding effect is greater than their positive externalities, which reduces the production efficiency of enterprises. Therefore, this paper proposes hypothesis 1.

#### Hypothesis 1

There is an inverted U-shaped relationship between industrial specialization agglomeration and UGLUE.

### Industrial diversification agglomeration and UGLUE

Industrial diversification agglomeration refers to the agglomeration of different industries in a specific area. Similar to industrial specialization agglomeration, it promotes UGLUE through the following three aspects (see Fig. 1): Firstly, financial externalities. The agglomeration of different companies in a specific area provides more types of final consumer goods and intermediate products, which not only reduces the production cost and transportation cost of enterprise, but also reduces the purchase cost of consumers. We call this capital externality as capital externality II. Secondly, technical externalities. The agglomeration of different industries in a specific area could improve the exchange of complementary knowledge and generation of new knowledge, reduce the cost of enterprise communication and learning, and form external economies of scale. The externality formed by knowledge spillover is called Jocabs technical externality. Thirdly, the externalities of labor market. In areas where industries are diversified and concentrated, when an industry is impacted, workers move to other industries more easily, thus avoiding the risk of unemployment. This kind of complementary labor market gives workers more choices and security, and improve output efficiency by promoting the agglomeration of a large amount of labor. We call this externality as complementary labor market externality. The three externalities jointly improve the production efficiency of enterprises, thereby improving the economic benefits of land. Therefore, this paper proposes hypothesis 2.

#### Hypothesis 2

Industrial diversification agglomeration can promote the improvement of UGLUE.

### Time lag effect of UGLUE

Figure 1 shows that UGLUE has time lag effect. Since land use is a dynamic and continuous process, the improvement of UGLUE in the previous period inevitably improve the current UGLUE by improving the social environment and economic development. Based on the above analysis, this paper proposes hypothesis 3.

#### Hypothesis 3

The UGLUE has a time lag effect.

## Research area, methods and data source

### Research area

**Figure 2:**
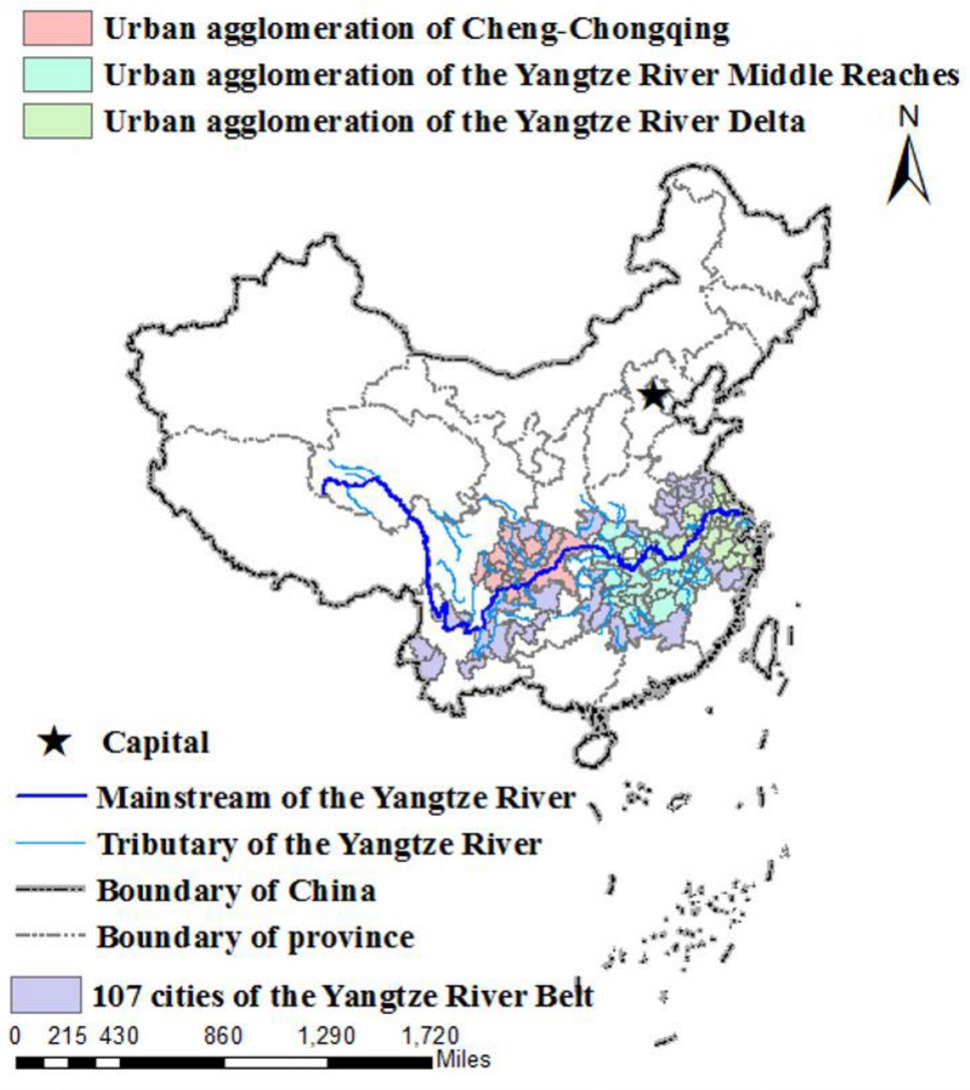
The geographic location of the YREB in China.

The YREB covers Shanghai, Jiangsu, Zhejiang, Anhui, Jiangxi, Hubei, Hunan, Chongqing, Sichuan, Guizhou, and Yunnan. It includes the Yangtze River Delta urban agglomerations (YRDUA), Yangtze River midstream urban agglomeration (YRMUA), and Chengdu-Chongqing urban agglomeration (CCUA). With a regional area of 2.05 million km^2^, the YREB runs through the eastern, central and western regions in China^32^. In 2019, the total GDP of YREB is 45.8 trillion yuan, accounting for 46.2% of the national GDP. The YREB plays a pivotal strategic support and leading role in the overall situation of stable economic growth in China^33^. At the same time, the contradiction between the shortage of land resources and economic growth in the YREB is very prominent. Therefore, this paper selects 107 cities in YREB as the research sample. The specific geographic locations are shown in Fig. 2. This article uses ARCGIS 10.2 version to draw the map. The URL link is http://demo.domain.com:6080/arcgis/services.

### Research methods

#### Global Malmquist–Luenberger index

UGLUE refers to the effective utilization degree of land elements under certain input of other elements. The green utilization of urban land mainly comes from three aspects: first, improve the utilization intensity of the existing actual input land, that is, increase the input intensity of other elements of the unit land area. Second, reduce the input of land in the production process to avoid excessive waste of land. Third, promote the optimal allocation of land elements among production units. Technical efficiency refers to the maximum degree that all factor inputs need to expand or shrink in equal proportion when all production units reach the production frontier. However, for production units with high technical efficiency, the factor allocation structure may not be reasonable. The land factors may still have the problem of under-input or over-input, resulting in the reduction of UGLUE.

Pastor and Lovell^34^ proposed a global index, which uses all the inspection periods of each decision-making unit as a benchmark to construct the production frontier. According to the current benchmark construction period t, the production possibility set reference set is defined as follows:

1 $$P_{C}^{t} (x^{t} ) = \left\{ {\left. {(y^{t} ,b^{t} )} \right|x^{t} {\kern 1pt} can{\kern 1pt} \, produce{\kern 1pt} \, b^{t} ,y^{t} } \right\}$$

The global benchmark is defined as: $P_{G} = P_{C}^{1} \, \cup \,P_{C}^{2} \, \cup \, \cdots \,P_{C}^{t}$, The subscripts C and G represent the current benchmark and the global benchmark respectively. The ML index of decision-making unit i is calculated based on the current reference benchmark:

2 $$ML^{S} (x^{t} ,y^{t} ,b^{t} ,x^{t + 1} ,y^{t + 1} ,b^{t + 1} ) = \frac{{1 + D_{C}^{S} (x^{t} ,y^{t} ,b^{t} )}}{{1 + D_{C}^{S} (x^{t + 1} ,y^{t + 1} ,b^{t + 1} )}}$$

Among them, the superscript S indicates two adjacent periods, t period and t + 1 period. The subscript C indicates the current benchmark, which is a simplified directional distance function. $ML^{s} > 1$, indicates that the productivity increases. $ML^{s} < 1$, indicates that the productivity decreases.

According to Hofmann et al.^35^, the GMLI is defined as follows:

3 $$GMLI^{t,t + 1} (x^{t} ,y^{t} ,b^{t} ,x^{t + 1} ,y^{t + 1} ,b^{t + 1} ) = \frac{{1 + D_{G}^{T} (x^{t} ,y^{t} ,b^{t} )}}{{1 + D_{G}^{T} (x^{t + 1} ,y^{t + 1} ,b^{t + 1} )}}$$

Among them, $D_{G}^{T} (x,y,b) = \max \left\{ {\alpha |(y - \alpha y,b - \alpha b) \in P_{G} (x)} \right\}$. $GMLI^{t,t + 1} > 1$ indicates that the productivity has increased. $GMLI^{t,t + 1} < 1$ indicates that the productivity decreases. The GMLI is further broken down as follows:

4 $$\begin{aligned} & GMLI^{t,t + 1} (x^{t} ,y^{t} ,b^{t} ,x^{t + 1} ,y^{t + 1} ,b^{t + 1} ) = \frac{{1 + D_{G}^{T} (x^{t} ,y^{t} ,b^{t} )}}{{1 + D_{G}^{T} (x^{t + 1} ,y^{t + 1} ,b^{t + 1} )}} \\ & \quad = \frac{{1 + D_{G}^{t} (x^{t} ,y^{t} ,b^{t} )}}{{1 + D_{G}^{t + 1} (x^{t + 1} ,y^{t + 1} ,b^{t + 1} )}} \times \left[ {\frac{{(1 + D_{G}^{T} (x^{t} ,y^{t} ,b^{t} ))/(1 + D_{C}^{T} (x^{t} ,y^{t} ,b^{t} ))}}{{(1 + D_{G}^{T} (x^{t + 1} ,y^{t + 1} ,b^{t + 1} ))/(1 + D_{C}^{T} (x^{t + 1} ,y^{t + 1} ,b^{t + 1} ))}}} \right] \\ & \quad = \frac{{TE^{t + 1} }}{{TE^{t} }} \times \left( {\frac{{BPG_{t + 1}^{t + 1} }}{{BPG_{t}^{t + 1} }}} \right) = EC_{t}^{t + 1} \times BPC_{t}^{t + 1} \\ \end{aligned}$$

Among them, TE is the change of technological progress. EC is the change of technological efficiency. The change of technological progress reflects the change of the highest technical level. The improvement of the highest technical level often requires the introduction and innovation of advanced technology, which often requires a large amount of investment. The change of technical efficiency reflects the gap with the highest technical level. Narrowing the gap with the highest technical level often requires improvements in internal management and governance structures. $BPG_{t}^{t + 1}$ is the “best practitioner gap” between the current period and overall technological frontier. $BPC_{t}^{t + 1}$ measures the changes in the “best practitioner gap” between two periods (technological changes). $BPC_{t}^{t + 1} \, > \, 1 \,$ indicates technological progress. $BPC_{t}^{t + 1} < 1$ indicates technology regress.

#### Econometric techniques of industrial agglomeration on UGLUE

In recent years, many scholars used the traditional SPM for empirical analysis, which is a basic measurement model suitable for panel data. Therefore, this article firstly uses the traditional SPM to analyze the impact of industrial agglomeration on UGLUE. The formula is:

5 $$\begin{aligned} \ln UGLUE_{it} & = \alpha_{0} + \alpha_{1} \ln RZI_{it} + \alpha_{2} \ln RZI_{it} *\ln RZI_{it} + \alpha_{3} \ln RDI_{it} + \alpha_{4} \ln EC_{it} \\ & \quad + \alpha_{5} \ln GDP_{it} + \alpha_{6} \ln TEC_{it} + \alpha_{7} \ln ROAD_{it} + \alpha_{8} \ln GOV_{it} + \varepsilon_{it} \\ \end{aligned}$$

Among them, ε is the disturbance term. i represents the city, i in this paper involves 107 cities in YREB. t represents the time, and the range of t in this paper is from 2007 to 2016. UGLUE is the explained variable, which represents the UGLUE. RZI and RDI are explanatory variables, representing industrial specialization agglomeration and industrial diversification agglomeration. EC is the industrial structure. GDP is the level of economic development. TEC is the level of technology. ROAD is the level of infrastructure. GOV is the degree of government intervention. $\alpha_{1}$ to $\alpha_{8}$ is the coefficient of each variable.

Formula (5) assumes that the UGLUE changes with the changes of various influencing factors in the current period. That is, there is no time lag effect. But in reality, land use often has a time lag effect. The previous level has a non-negligible impact on the current results. Therefore, this paper selects the dynamic panel model for empirical analysis. However, there is often a two-way causal relationship between industrial agglomeration and UGLUE, which may cause endogenous bias. For example, cities with higher UGLUE levels tend to have higher levels of economic development, which promotes industrial agglomeration in this city. Therefore, this paper adopts the method of system GMM for regression analysis of dynamic panel model. Compared with mixed OLS, system GMM can make full use of sample information, select appropriate lag terms as instrumental variables^36^. It can effectively solve the endogeneity problem between industrial agglomeration and UGLUE. Based on the above analysis, this paper introduces the first-order lag term of UGLUE on the basis of formula (5). The DPM is as follows:

6 $$\begin{aligned} \ln UGLUE_{it} & = \beta_{0} + \tau \ln UGLUE_{i(t - 1)} + \beta_{1} \ln RZI_{it} + \beta_{2} \ln RZI_{it} \times \ln RZI_{it} + \beta_{3} \ln RDI_{it} \\ & \quad + \beta_{4} \ln EC_{it} + \beta_{5} \ln GDP_{it} + \beta_{6} \ln TEC_{it} + \beta_{7} \ln ROAD_{it} + \beta_{8} \ln GOV_{it} + \varepsilon_{{{\text{it}}}} \\ \end{aligned}$$

Among them, $\tau$ is the first-order lag coefficient of UGLUE, reflecting the time lag effect of UGLUE.

### Variable description

#### Explained variable

The GMLI is used to measure the UGLUE of 107 cities in YREB. According to existing research^37^, the following core evaluation index of UGLUE are selected (see Table 1). Regarding input indicators, we mainly choose land element input M, labor element input L, and capital element input K as input indicators. Regarding output indicators, we choose the added value of the secondary and tertiary industries in the municipal area as the expected output, and use the GDP deflator to convert it into a comparable value. At the same time, pollution indicators such as industrial wastewater emissions, industrial sulfur dioxide emissions, and industrial smoke (dust) emissions are selected as undesired output. Since the GMLI reflects the growth rate of UGLUE, this paper assumes that the GMLI in 2006 is 1, and then multiplies the calculated GMLI year by year to obtain the development level of UGLUE in each city from 2007 to 2016.

**Table 1:** Input and output index.

Index	Variable	Description
Input Index	M	Area of construction land in municipal district (km^2^)
	K	Investment in fixed assets of municipal district (100 million yuan)
	L	Employees of secondary and tertiary industries in municipal district (10 thousand people)
Expected output Index	GDP	Added value of secondary and tertiary industries in municipal district (10 thousand yuan)
Unexpected output Index	WW	Industrial wastewater discharge (10 thousand tons)
	SO_2_	Industrial sulfur dioxide emissions (ton)
	SD	Industrial smoke (dust) emissions (ton)

#### Explanatory variables

Industrial specialization index ZI is usually used to measure the specialization level of urban industries. The specialization index is represented by the share of the employment of the industry in the total employment of the city:

7 $$ZI_{i} = Max_{j} (S_{ij} )$$

Nextly, we use the relative specialization index to make a horizontal comparison of the specialization level between different cities:

8 $$RZI_{i} = Max(S_{ij} /S_{j} )$$

The most common measure of the level of industrial diversification is the Herfindahl–Hirschman Index (HHI). For city i, the HHI is the sum of the square sum of employment shares of all industries in the city. The diversification index is the reciprocal of the HHI:

9 $$DZ_{i} = \frac{1}{{\sum\limits_{j} {S_{ij}^{2} } }}$$

The expression of relative diversification index is as follows:

10 $$RDI_{i} = {1 \mathord{\left/ {\vphantom {1 {\sum\limits_{j} {\left| {S_{ij} - S_{j} } \right|} }}} \right. \kern-0pt} {\sum\limits_{j} {\left| {S_{ij} - S_{j} } \right|} }}$$

Among them, S_ij_ is the employment proportion of j industry in city i, and S_j_ is the proportion of the total employment of the national j industry. The greater value of RZI and RDI, the higher level of industrial specialization and diversification.

#### Control variables

Regarding control variables, we choose the following variables as control variables.

Industrial structure (EC): The continuous optimization of industrial structure promotes the improvement of UGLUE through three aspects: saving land, increasing land income and promoting the optimal allocation of land resources. This paper selects the added value of the tertiary industry as a percentage of GDP (take the logarithm) to express.

Technological level (TEC): The higher the technological innovation level of a city is, the more it promotes the use of input elements and the transformation of innovation results, thereby improving the UGLUE. This paper selects the proportion of science and technology expenditure to fiscal expenditure (take the logarithm) to represent.

Economic development level (GDP): The continuous economic development promote the rational allocation of various production factors and increase the level of urban land output, thereby improving the UGLUE. This paper selects GDP per capita (take the logarithm) to express.

Road infrastructure level (ROAD): The continuous improvement of infrastructure reduces transportation costs and transaction costs, and promotes communication externalities between producers, consumers, and between producers and consumers. This paper selects the average road area per capita (take the logarithm) to express.

Government behavior (GOV): Fiscal expenditure is an important means for the government to carry out macro-control. Appropriate fiscal expenditure makes up for market shortages, improves factor flow and resource allocation efficiency, and realizes positive economic externalities. This paper selects the proportion of fiscal expenditure to GDP (take the logarithm) to express. We can see the meaning of specific variables from Table 2.

**Table 2:** The descriptive statistics of variables.

Variable name	Characterization	Obs	Mean	Std	Min	Max
UGLUE	Global malmquist–luenberger index (take the logarithm)	1070	2.3055	0.3615	− 0.4638	4.1020
RZI	$RZI_{i} = Max(S_{ij} /S_{j} )$	1070	3.1889	0.4232	2.5341	5.4435
RDI	$$RDI_{i} = {\raise0.7ex\hbox{$1$} \!\mathord{\left/ {\vphantom {1 {\sum\nolimits_{j} {\left| {S_{ij} - S_{j} } \right|} }}}\right.\kern-0pt} \!\lower0.7ex\hbox{${\sum\nolimits_{j} {\left| {S_{ij} - S_{j} } \right|} }$}}$$	1070	3.1181	0.3418	1.9391	3.9935
EC	The proportion of added value of tertiary industry to GDP (take the logarithm)	1070	3.7036	0.2431	2.9413	4.3383
TEC	The proportion of science and technology expenditure to financial expenditure (take the logarithm)	1070	10.2961	0.6976	8.3770	12.2012
GDP	Per capita GDP (take the logarithm)	1070	2.5371	0.8136	0.4399	5.0921
ROAD	Per capita road area (take the logarithm)	1070	2.2303	0.5917	− 0.5276	4.2716
GOV	The proportion of fiscal expenditure to GDP (take the logarithm)	1070	1.9753	0.3996	0.7208	3.1239

### Data source

The object of this thesis is the 107 cities in YREB from 2007 to 2016. The urban construction land area data comes from the "China Urban Construction Statistical Yearbook", and the rest of the index data all come from the "China City Statistical Yearbook". The URL link is https://www.cnki.net/. In order to maintain the integrity of the data, this article uses the average method to fill in the missing values. In addition, because Chaohu City began to be under the jurisdiction of Hefei City in 2011, Bijie City and Tongren City in Guizhou Province only became prefecture-level cities in 2011. The three cities and Pu'er City are taken from the sample to maintain the continuity of data.

## The impact of industrial agglomeration on UGLUE

### The changing trend of UGLUE

**Figure 3:**
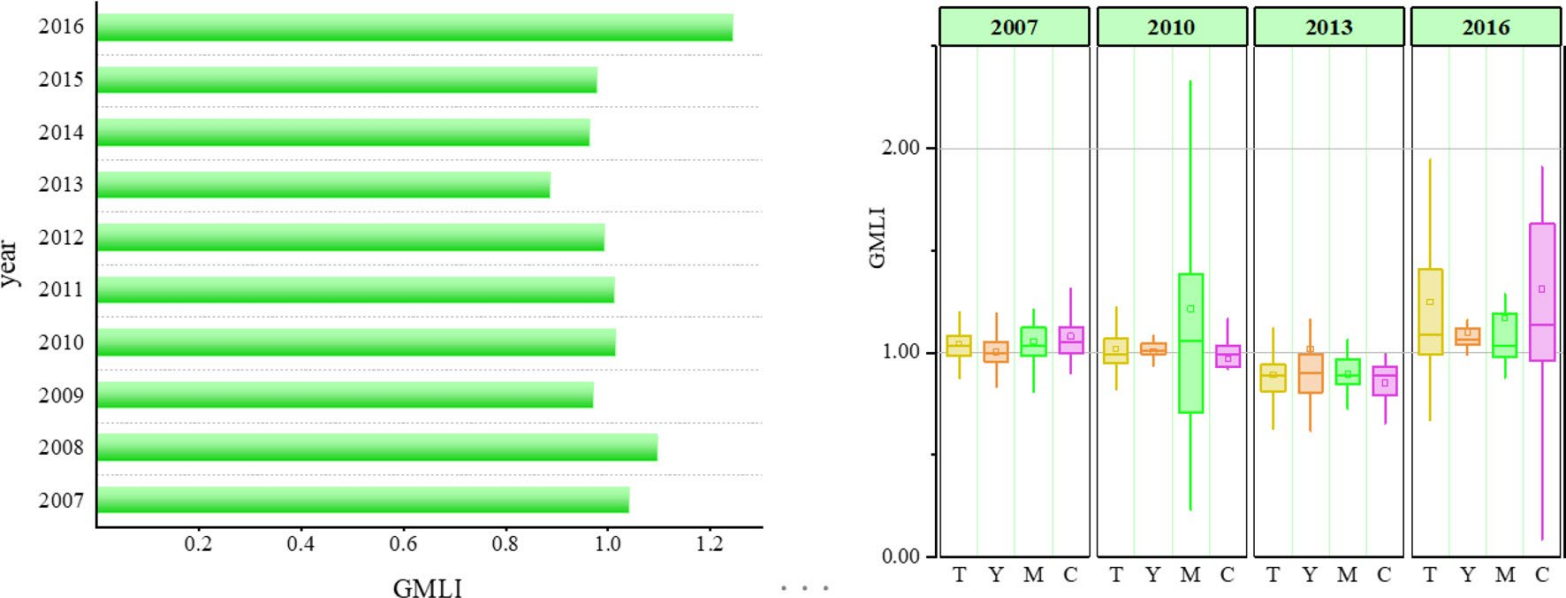
Average GMLI of UGLUE in YREB from 2007 to 2016.

**Figure 4:**
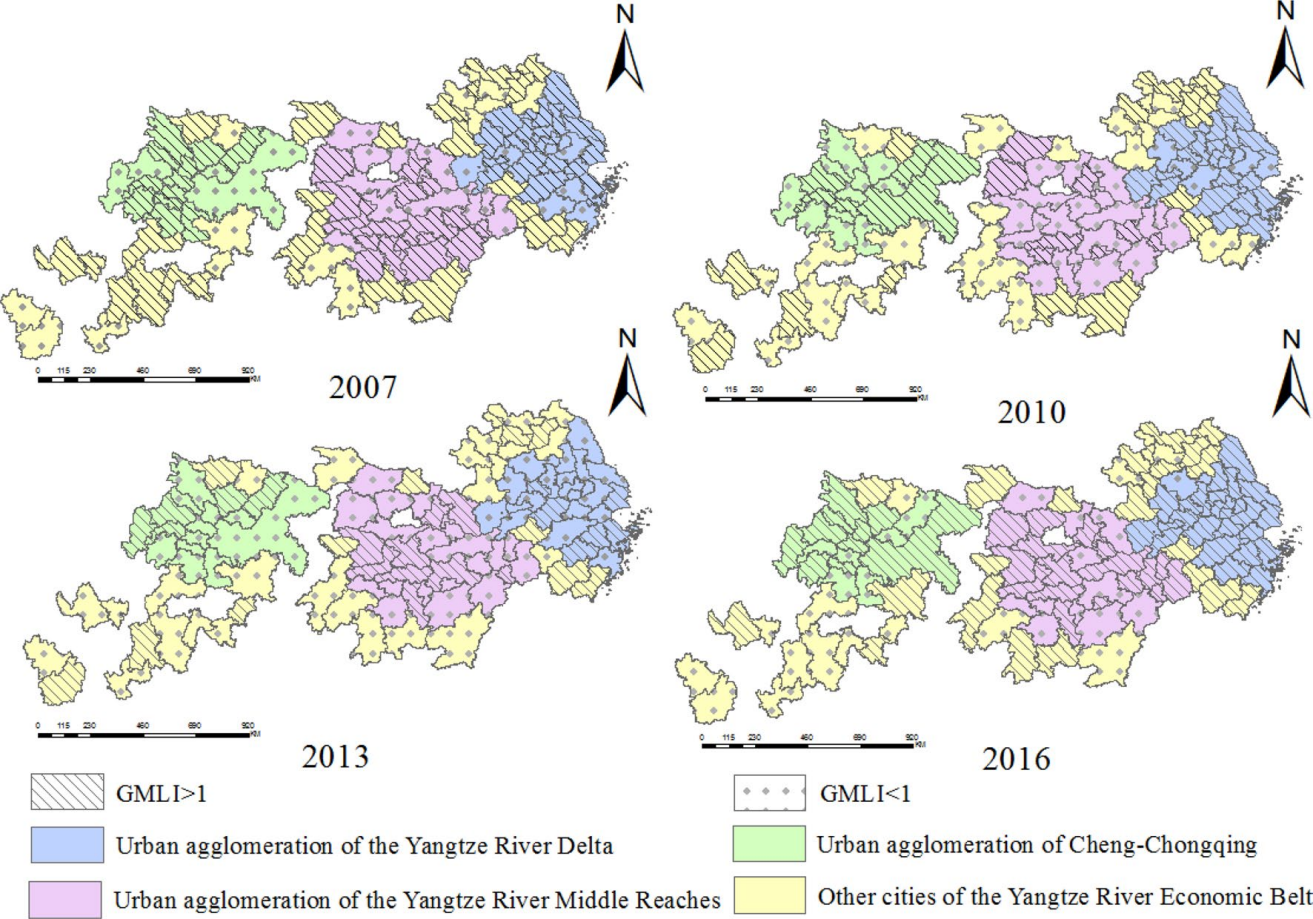
Spatial distribution map of GMLI in 2007, 2010, 2013 and 2016.

This article uses ARCGIS 10.2 version to draw Fig. 3 and Fig. 4. The URL link is http://demo.domain.com:6080/arcgis/services. Figure 3 shows the GMLI trend of UGLUE in YREB from 2007 to 2016. The GMLI of YREB fluctuates between 0.890 and 1.242. The GMLI in 2009, 2012, 2013, 2014 and 2015 is less than 1, indicating that the UGLUE has been reduced by about 3%. The average value of GMLI in 2011 is 1.030, indicating that the utilization of land investment is relatively high. But it declines after 2012. The average GMLI in 2015 is 0.959, indicating that the UGLUE is declining at an average annual rate of 4.1%. The average value of GMLI in 2016 is 1.242, indicating that the UGLUE has been greatly improved. At the same time, we find that the GMLI fluctuation ranges of the YRDUA, YRMUA and CCUA is between 0.914 to 1.108, 0.893 to 1.214, and 0.853 to 1.310, respectively. Among them, the YRDUA has the lowest average GMLI, which is 0.909. The CCUA comes next with 0.935. The average GMLI of YRMUA is 0.958, which shows that the UGLUE in three urban agglomerations has declined to varying degrees. The waste of land resources is serious.

Observing Fig. 4, we can see that there are many cities with GMLI less than 1 in 2013 and a few cities with GMLI less than 1 in 2016. This shows that there are more and more cities where UGLUE has increased in YREB. The UGLUE is gradually moving to the production frontier.

### Baseline estimation results

In theory, land use is a dynamic process. The neglect of time lag effect also seriously affects the accuracy of the estimation results. Therefore, we use DPM that combines time lag effect for empirical analysis. We use the following four models at the same time: SPM and DPM. The hybrid OLS is used to estimate the SPM, and the system GMM is used to estimate the DPM. The system GMM model not only considers the dynamic effect of UGLUE, but also solves the endogenous problem to a certain extent. The estimation results are shown in Table 4. Columns (1) and (2) report the estimation results of SPM. Columns (3) and (4) report the estimation results of DPM.

In Table 3, the estimation results of AR(1) and AR(2) show that at the significance level of 10%, there is a first-order autocorrelation for the difference of the perturbation term, but no second-order autocorrelation, indicating that the model is reasonably set. Hansen test results do not reject the null hypothesis that instrumental variables are valid, which indicates that the selection of instrumental variables is reasonable. In terms of the core explanatory variable, the estimated coefficient of industrial specialization agglomeration primary term is 0.0151, which passes the 1% significance level. The estimated coefficient of industrial specialization agglomeration quadratic term is -0.0265, which shows that there is an inverted U-shaped relationship between industrial specialization agglomeration and UGLUE. The estimated coefficient of industrial diversification agglomeration is 0.0778, which passes the 1% significance level. For every 1% increase in the level of industrial diversification agglomeration, the UGLUE increases by 0.0778%, which shows that industrial diversification agglomeration promotes the improvement of UGLUE.

**Table 3:** Estimated results of SPM and DPM.

	SPM		DPM	
	(1)	(2)	(3)	(4)
L.lnUGLUE			0.858***	0.858***
			(0.0090)	(0.0108)
lnRZI	0.0077		0.151***	
	(0.256)		(0.0531)	
lnRZI*lnRZI	− 0.0019		− 0.0265***	
	(0.0353)		(0.0074)	
lnRDI		0.0487		0.0778***
		(0.0336)		(0.0175)
lnEC	0.297***	0.312***	0.168***	0.184***
	(0.0498)	(0.0492)	(0.0103)	(0.0363)
lnGDP	0.129***	0.131***	0.0666***	0.0722***
	(0.0254)	(0.0253)	(0.0053)	(0.0082)
lnTEC	0.0246	0.0198	0.0073	− 0.00062
	(0.0205)	(0.0206)	(0.0080)	(0.0068)
lnROAD	− 0.112***	− 0.118***	− 0.0816***	− 0.0848***
	(0.0235)	(0.0238)	(0.0053)	(0.0076)
lnGOV	− 0.146***	− 0.144***	− 0.0559***	− 0.0686***
	(0.0351)	(0.0345)	(0.0082)	(0.0196)
AR(1)			0.000	0.000
AR(2)			0.413	0.419
Hansen test			0.322	0.330
Cons	0.681	0.151	− 0.789***	− 0.858***
	(0.504)	(0.3083)	(0.112)	(0.1622)
*N*	1070	1070	963	963
R^2^	0.072	0.0685		

Regarding the time lag effect of UGLUE, the estimated coefficient of the first-order lag term of UGLUE is significantly positive at the 1% level, indicating that UGLUE has time lag effect. If the UGLUE is at a relatively high level. It will continue to increase in the next phase. At the same time, it also shows that it is urgent to improve the UGLUE, otherwise the difficulty will become greater.

Regarding the control variables, the impact of industrial structure on UGLUE is positive, which passes the 1% significance level. Because the optimization of industrial structure is conducive to saving land, increasing land revenues, and promoting the optimal allocation of land resources, thereby having a positive impact on UGLUE. The level of economic development has a positive impact on UGLUE. The improvement of the level of economic development can increase the economic benefits of land and promote the improvement of UGLUE. Therefore, cities with low levels of economic development can improve UGLUE through economic development. The impact of science and technology on UGLUE is positive, but not significant. This is because the proportion of science and technology expenditure is low, which is not enough to promote the full use of factors, so technological progress cannot improve UGLUE. The estimated coefficient of infrastructure level is significantly negative at the 1% level. This may be because the excessive loss of roads and maintenance costs increase transportation costs, thereby restricting the free circulation of various production factors among larger areas.

### Robustness test

Outliers will cause deviations in the estimated results. In order to ensure the accuracy of the estimated results, this paper carries out tailing reduction treatment at 1% level for all variables. In addition, the index of employment in industrial agglomeration is replaced by the total output value of the industry, and the industrial specialization index and diversification index are re-measured.

The estimation results are shown in Table 4. Columns (1) and (2) report the estimation results after 1% of the variables are shortened, and Columns (3) and (4) report the results of the estimation of the re-measure of the core explanatory variables. The latter estimation results are similar to the previous. The impact of industrial specialization agglomeration on UGLUE is significantly negative. The impact of industrial diversification agglomeration on UGLUE is significantly positive, indicating that the estimation results are robust.

**Table 4:** Estimation results of robustness test.

	(1)	(2)	(3)	(4)
L.lnUGLUE	0.846***	0.817***	0.767***	0.763***
	(0.0117)	(0.0105)	(0.0078)	(0.0086)
lnRZI	0.0525***		0.0586***	
	(0.0086)		(0.0049)	
lnRZI*lnRZI	− 0.0067***		− 0.0336***	
	(0.0009)		(0.0085)	
lnRDI		0.0642***		0.122***
		(0.0188)		(0.0133)
lnEC	0.168***	0.178***	0.523***	0.580***
	(0.0304)	(0.0240)	(0.0265)	(0.0255)
lnGDP	0.0779***	0.0820***	0.107***	0.173***
	(0.0111)	(0.0130)	(0.0100)	(0.0116)
lnTEC	− 0.0044	− 0.0074*	0.0430***	− 0.0339**
	(0.0070)	(0.0043)	(0.0100)	(0.0142)
lnROAD	− 0.0871***	− 0.0809***	− 0.124***	− 0.136***
	(0.0135)	(0.0110)	(0.0142)	(0.0147)
lnGOV	− 0.0565***	− 0.0923***	− 0.283***	− 0.327***
	(0.0171)	(0.0270)	(0.0156)	(0.0131)
AR(1)	0.000	0.000	0.000	0.000
AR(2)	0.446	0.453	0.402	0.510
Hansen test	1.000	1.000	1.000	1.000
Cons	− 0.458***	− 0.691***	− 0.938***	− 1.977***
	(0.129)	(0.153)	(0.113)	(0.150)
*N*	963	963	963	963

### Heterogeneity analysis

The three major urban agglomerations in YREB include the YRDUA, YRMUA, and CCUA. The size, economic structure and industrial layout of the urban agglomerations differ significantly. The development of the manufacturing industry in YRDUA is relatively mature. The industrial layout of YRMUA is dominated by the secondary industry. Although the proportion of the tertiary industry in CCUA is equivalent to that of the YRDUA, the proportion of manufacturing is relatively low. Many differences among urban agglomerations may bring about differences in the effects of industrial agglomeration on UGLUE.

This paper uses a DPM to estimate the impact of industrial agglomeration on UGLUE of the three major urban agglomerations in the YREB. The results are shown in Table 5. Industrial specialization agglomeration in the YRDUA has an inverted U-shaped effect on UGLUE. The estimated coefficient of industrial diversification agglomeration is -0.0275, but it is not significant. Industrial specialization agglomeration has a positive U-shaped effect on UGLUE in the YRMUA. The estimated coefficient of industrial diversification agglomeration is -0.0122, but it is not significant. The impact of industrial agglomeration on UGLUE in the CCUA is not significant. This may be related to the level of economic development of the three major urban agglomerations. The high level of economic development in the YRDUA attracts a large number of similar industries to gather here. The excessive gathering of enterprises produces crowding effect, forms malicious competition and squeezes the profit space of enterprises, which reduce the production efficiency of enterprises. However, the economic development level in the CCUA is not high. The degree of industrial agglomeration is not enough to significantly affect the UGLUE.

**Table 5:** Estimated results of regional heterogeneity.

	YRDUA		YRMUA		CCUA	
	(1)	(2)	(3)	(4)	(5)	(6)
L.lnUGLUE	0.670***	0.729***	0.464***	0.514***	0.524	0.434
	(0.164)	(0.157)	(0.0708)	(0.108)	(0.426)	(0.336)
lnRZI	0.943**		− 1.260*		0.990	
	(0.460)		(0.671)		(1.698)	
lnRZI*lnRZI	− 0.158**		0.198**		− 0.125	
	(0.0647)		(0.0997)		(0.256)	
lnRDI		− 0.0275		− 0.0122		− 0.0798
		(0.122)		(0.130)		(0.0913)
lnEC	− 0.0409	0.0751	0.191	0.101	− 0.0776	− 0.290
	(0.174)	(0.251)	(0.179)	(0.199)	(0.403)	(0.414)
lnGDP	0.0014	− 0.0694	0.0655	− 0.0105	− 0.0345	0.0331
	(0.0559)	(0.115)	(0.0558)	(0.0546)	(0.347)	(0.227)
lnTEC	0.0306	0.0564	− 0.104**	− 0.0439	0.0939	− 0.0814
	(0.0640)	(0.108)	(0.0466)	(0.0435)	(0.164)	(0.122)
lnROAD	− 0.0276	− 0.0945	0.00788	0.0359	− 0.292	− 0.100
	(0.0585)	(0.128)	(0.0973)	(0.109)	(0.195)	(0.228)
lnGOV	− 0.141	0.0558	− 0.145*	− 0.0392	0.0330	− 0.293
	(0.170)	(0.197)	(0.0805)	(0.103)	(0.619)	(0.605)
AR(1)	0.000	0.000	0.000	0.000	0.000	0.000
AR(2)	0.270	0.280	0.248	0.229	0.481	0.404
Hansen test	1.000	1.000	1.000	1.000	1.000	1.000
Cons	0.0520	0.933	2.694	1.104	0.0488	3.736
	(1.973)	(1.647)	(1.640)	(0.944)	(4.896)	(2.724)
*N*	234	234	252	252	144	144

## Discussion

The impact of industrial specialization agglomeration on the UGLUE shows an inverted U-shape, which is in line with the hypothesis 1 of this paper. The crowding effect of factor market and product market is greater than the externality generated is the main reason for this result. On the one hand, gatherings of similar companies in specific areas intensify the competition among companies for various resources, resulting in rising prices of production factors. On the other hand, because there are too many homogenized products in the market, companies adopt low-price sales strategies to improve their competitive advantages, which severely squeeze the company's profit margins, reduce the company’s investment in innovative technologies and output efficiency. The phenomenon of industrial homogeneity in the YREB is very serious. For example, there are more than 20 logistics parks in 8 cities along the Yangtze River in Jiangsu Province, which forms crowding effect of industrial agglomeration and hinders the coordinated development of the overall industry and the improvement of UGLUE.

The impact of industrial diversification agglomeration on UGLUE is significantly positive, which is in line with the hypothesis 2 of this paper. The main reason for this result is the dominance of capital, technology and complementary labor market externalities. Firstly, the existence of capital externalities not only reduce the production costs of enterprises, but also reduce people's purchase costs. Secondly, the technological externalities promote the exchange of complementary knowledge and technology between enterprises, which reduce the cost of enterprise learning and communication, thereby improving the level of technological innovation and production efficiency of enterprises. Thirdly, the complementary labor market externalities give labor more employment opportunities and security, promote labor agglomeration and enterprise production efficiency. The «“Thirteenth Five-Year” National Strategic Emerging Industry Development Plan» issued by the State Council pointed out that it is necessary to accelerate the creation of strategic emerging industries development sources, develop characteristic industrial clusters, and form a new pattern of industrial agglomeration development. In order to actively respond to national policies, Sichuan Province has vigorously promoted the development of the “Double Seven and Double Five” industries, focusing on cultivating 14 industries such as energy-saving and environmental protection equipment, aviation and gas turbines, information security, rail transit, modern finance, and health and elderly care. The echelon growth structure of industrial development begins to take shape, which increases the economic benefits of the land.

## Conclusions and policy recommendations

### Conclusions

This paper uses GMLI to measure the UGLUE of 107 cities in the YREB from 2007 to 2016, and uses a DPM to empirically analyze the impact of industrial specialization agglomeration and industrial diversification agglomeration on UGLUE. The conclusions are as follows: (1) The UGLUE in the YREB shows a downward trend on the whole. There is still a large room for improvement. At the same time, the number of cities where UGLUE was improved increased significantly. (2) Different industrial agglomeration modes have different impacts on UGLUE. Regarding industrial specialization agglomeration, there is an inverted U-shaped relationship between industrial specialization agglomeration and UGLUE. Low level of industrial specialization agglomeration can improve UGLUE. With the increase of industrial specialization agglomeration level, the impact of industrial specialization agglomeration on UGLUE changes from positive to negative. As for industrial diversification agglomeration, industrial diversification agglomeration can have a positive impact on UGLUE through capital externalities, technology externalities and labor market externalities. (3) Industrial agglomeration has a heterogeneous effect on UGLUE in different urban agglomerations. The effect of industrial specialization agglomeration on UGLUE in the YRDUA is inverted U-shaped, while the effect of industrial diversification agglomeration on UGLUE is not significant. Industrial specialization agglomeration in the YRMUA has a positive U-shaped effect on UGLUE. The effect of industrial specialization agglomeration on UGLUE is also not significant. However, industrial agglomeration in CCUA has no significant effect on UGLUE.

### Policy recommendations

Firstly, give full play to the advantages of professional agglomeration and avoid extensive use of land. The effect of industrial specialization agglomeration on UGLUE is nonlinear. Low level of specialization agglomeration can improve UGLUE. With the continuous improvement of industrial specialization agglomeration, the influence of industrial specialization agglomeration on UGLUE changes from positive to negative. Therefore, attention should be paid to the industrial specialization agglomeration process to give full play to the enhancement effect of industrial specialization agglomeration on UGLUE. Guizhou and Yunnan provinces can provide material guarantee for industrial specialization by establishing transportation networks and supporting facilities for related industries. At the same time, the entrance threshold of enterprises prone to heavy pollution should be raised. The production technology transformation of enterprises with high pollution should be forced by measures such as increasing taxes to improve UGLUE.

Secondly, raise the level of industrial diversification and agglomeration and promote the effective use of land. On one hand, it is necessary to strengthen the construction of investment platform, actively undertake industrial transfer in developed regions, expand production scale, and avoid excessive agglomeration of single industrial factors in this region. At the same time, local entrepreneurs should be strongly supported to carry out innovative and entrepreneurial projects, which can satisfy the diversified demand of consumers. On the other hand, the theory of comparative advantage and the development of industrial diversification should be combined to prevent the emergence of a “dominant stock” phenomenon in a certain industry in the region, thereby promoting the diversified development of local industries, thus improving the UGLUE.

Thirdly, formulate differentiated industrial agglomeration policies for different urban agglomerations. Industrial agglomeration in different urban agglomerations has different impacts on UGLUE. Therefore, for different urban agglomerations, local governments should vigorously develop advantageous industries and formulate industrial development policies according to local conditions. For the YRDUA, emerging industry clusters should be built. It is necessary to rationally embed emerging industries into the local industrial chain, which will form complementary advantages with other service industries and promote the diversified development of service industries. For the YRMUA, it is necessary to actively create conditions to promote the professional agglomeration of service industry. The proportion of information transmission, scientific research and technical services in the economic structure of service industries should be expanded, which will further strengthen the role of professional agglomeration of service industries in promoting UGLUE. For the CCUA, we should pay attention to infrastructure construction and investment to provide good material guarantee for industrial agglomeration. Strengthen the construction of roads, railways and other transportation infrastructure. Promote the close links between cities and industries, and avoid the occurrence of regional industrial monoculture.

## Data Availability

The data that support the findings of this study are available from [www.cnki.net] but restrictions apply to the availability of these data, which were used under license for the current study, and so are not publicly available. Data are however available from the authors upon reasonable request and with permission of [www.cnki.net]. Those wishing to request data from this study can contact Ping Han.
